# HDAC6 Mediates Macrophage iNOS Expression and Excessive Nitric Oxide Production in the Blood During Endotoxemia

**DOI:** 10.3389/fimmu.2020.01893

**Published:** 2020-08-20

**Authors:** Yan Wang, Ke Wang, Jian Fu

**Affiliations:** ^1^Department of Respiratory and Critical Care Medicine, The Second Hospital of Jilin University, Changchun, Jilin, China; ^2^Department of Toxicology and Cancer Biology, College of Medicine, University of Kentucky, Lexington, KY, United States

**Keywords:** sepsis, inflammation, macrophage, nitric oxide, microtubule

## Abstract

Excessive nitric oxide (NO) production and NO-mediated nitrative stress contribute to vascular dysfunction, inflammation, and tissue injury in septic shock. New therapeutic targets are urgently needed to provide better control of NO level during septic shock. In the present study, we investigated the role of HDAC6 in the regulation of NO production and nitrative stress in a mouse model of endotoxin-induced septic shock. HDAC6 deficient mice and a specific HDAC6 inhibitor were utilized in our studies. Our data clearly indicate that HDAC6 is an important mediator of NO production in macrophages. HDAC6 mediates NO production through the regulation of iNOS expression in macrophages. HDAC6 up-regulates iNOS expression in macrophages by modulating STAT1 activation and IRF-1 expression. HDAC6 inhibition potently blocked endotoxin-induced STAT1 activation and iNOS expression in macrophages. Furthermore, HDAC6 contributes to excessive NO production and nitrotyrosine level in the blood and promotes iNOS expression in the lung tissues during septic shock. Our data reveal a novel HDAC6/STAT1/iNOS pathway that mediates excessive NO production and nitrative stress in septic shock.

## Introduction

Sepsis is a life-threatening disease that is defined as multi-organ dysfunction caused by dysregulated host responses to infection (1–5). Sepsis remains a major health problem worldwide (1, 4, 5). Septic shock, which is characterized by hypotension and hyporeactivity to vasoconstrictors, is a main cause of high mortality in sepsis patients (5–8). Septic shock leads to reduced blood and oxygen flow to vital organs, which eventually causes multi-organ failure and death (5–8). Endotoxemia is a major factor in the pathogenesis of septic shock (2, 3). Endotoxemia is caused by elevated levels of circulating bacterial endotoxin (2, 3), a lipopolysaccharide (LPS) component of the Gram-negative bacterial outer membrane. Endotoxemia has been detected in the critically ill sepsis patients (2, 3).

Nitric oxide (NO) has been identified as a key endogenous regulator of vascular tone (5–9). It is now well-established that excessive NO production contributes to vascular dysfunction during septic shock including vasodilation, vascular hyporeactivity, and vascular hyperpermeability (5–9). The stable NO metabolites nitrite and nitrate have been used as the indicators of NO production (5–9). The levels of the NO metabolites are increased during septic shock (5–9).

NO can also react with superoxide anion (O_2^-^_) to form highly cytotoxic oxidant peroxynitrite (ONOO^−^) (8–10). Nitrative stress is induced by peroxynitrite oxidation of lipids, proteins, and nucleic acids (8–10). Nitrative stress causes cytotoxicity in tissues and contributes to multi-organ injury in sepsis such as endothelial injury and vascular dysfunction (8–10).

NO is generated by nitric oxide synthases (NOS) through metabolism of L-arginine (9, 11, 12). The constitutively expressed endothelial NOS (eNOS) and neuronal NOS (nNOS) are thought to contribute to the physiological levels of NO (9, 11, 12). The inducible nitric oxide synthase (iNOS) is responsible for a large production of NO during inflammatory responses and disease states (9, 11, 12). iNOS can be expressed by a variety of cells and tissues including macrophages and neutrophils (9, 11, 12). iNOS expression in leukocytes is induced during sepsis (6, 8, 9, 13), which leads to high NO production in septic shock (6, 8, 9, 13). The NO generation through iNOS induction is one of the host defense mechanisms against bacterial infection (6, 8, 9, 13). However, excessive iNOS induction can lead to shock and tissue damage (6, 8, 9, 13).

iNOS expression is mainly regulated at the transcriptional level (8, 9, 12, 13). Activation of transcription factors such as nuclear factor κB (NFκB) and Signal transducer and activator of transcription 1 (STAT1) are needed for the maximal iNOS expression (8, 9, 12, 13). The active transcriptional factors bind to the promoter region of iNOS gene to induce iNOS expression. STAT-1 activation can also induce the expression of other regulatory factors such as interferon regulatory factor-1 (IRF-1) (9, 12, 13). IRF-1 then binds to the interferon-regulatory binding site at the iNOS promoter and facilitates iNOS expression (9, 12, 13).

HDAC6 (histone deacetylase 6) is a member of class II histone deacetylase that modulates the dynamics of protein acetylation and deacetylation (14–16). HDAC6 is mostly localized in the cytoplasm (14, 15). HDAC6 regulates many cellular responses and cell signaling pathways including stress responses, cell proliferation, inflammatory responses, apoptotic signaling, and transcription (14–16). However, the role of HDAC6 in iNOS expression and NO production has not been studied. In the present study, we investigated the role of HDAC6 in iNOS expression and nitric oxide production in macrophages. We also investigated the role of HDAC6 in excessive NO production and nitrative stress in endotoxin-induced septic shock.

## Materials and Methods

### Reagents

β-actin (Cat#5125), GAPDH (Cat#8884), IRF-1 (Cat#8478), iNOS (Cat#13120), p-STAT1(Y701) (Cat#7649), STAT1 (Cat#9172) and acetyl-α-tubulin (Lys40) (Cat#5335), HDAC6 (Cat#7612) antibodies were purchased from Cell Signaling Technology (Danvers, Massachusetts). Lipopolysaccharide (LPS) from Escherichia coli 0111:B4 (Cat# L4391) was purchased from Sigma Aldrich (St. Louis, Missouri). CAY10603 (Cat# S7596) was obtained from Selleck Chemicals (Houston, Texas).

### Mouse Model of Endotoxemia

All experiments and animal care procedures conform to the Guide for the Care and Use of Laboratory Animals and were approved by the Institutional Animal Care and Use Committee of the University of Kentucky. Wild type C57BL/6J mice (Stock No: 000664) and HDAC6 knockout C57BL/6J mice (Stock No:029318) were purchased from The Jackson Laboratory (Bar Harbor, Maine). The CRISPR/Cas9-generated HDAC6 knockout C57BL/6J mice carry a HDAC6 gene knock-out mutation. 9- to 12-week-old sex and age-matched wild type C57BL/6J mice and HDAC6 knockout C57BL/6J mice were used in the studies. Endotoxemia was induced by IP injection of LPS (7.5 mg/kg body weight). Experiments were terminated 6 h after LPS challenge. Blood and lung tissues were collected for the assays.

### Isolation of Mouse Peritoneal Macrophages

Mouse peritoneal macrophages were isolated as described previously (17). Briefly, after euthanasia, the abdomen skin of each mouse was socked with 70% alcohol. A small incision was made to expose the intact peritoneal wall. Five milliliters of ice-cold RPMI 1640 medium was injected into the peritoneal cavity. After gentle massage, peritoneal fluid was harvested and centrifuged at 500 g for 10 min at 4°C. The cells were then resuspended and cultured in RPMI 1640 containing 10% Fetal Bovine Serum. Peritoneal cells were allowed to adhere to the cell culture plates for 4 h at 37°C in 5% CO_2_ and 95% air. The plates were then washed to remove non-adherent cells. The attached macrophages were used for the experiments. Differential cell staining was performed with a Wright-Giemsa stain set (astraldiagnostics; Item# 5585). Differential cell counts showed >97% macrophages. The cell viability was >98% as determined by trypan blue dye exclusion.

### *In vitro* and *in vivo* Assays for Nitric Oxide Production

Nitric oxide production in peritoneal macrophages was examined by nitrite quantitation using Griess Reagent Kit (Cat# G7921; Thermo Fisher Scientific; Grand Island, New York) according to the manufacturer's protocol. Briefly, peritoneal macrophages were divided equally and plated in the 96-well plate. After LPS and CAY10603 treatment, the cell culture supernatant was collected and mixed with Griess Reagent. The mixture was incubated for 30 min at room temperature. The absorbance was measured at 548 nm using a plate reader. Nitrite concentration was then calculated according to the standard curve.

*In Vivo* blood nitric oxide levels were examined by nitrite/nitrate quantitation using the Nitric Oxide Colorimetric Assay Kit (Cat#K262; Biovision; Milpitas, California) according to the manufacturer's protocol. Briefly, mouse plasma was collected and filtered through a 10 KDa cutoff filter, then the Nitrate Reductase mixture and the enzyme cofactor were added to the plasma in the plates. The plates were incubated at room temperature to convert nitrate to nitrite. The enhancer and Griess Reagent were then added. After the color was developed for 10 min at room temperature, the absorbance was measured at 540 nm in a plate reader.

### *In vivo* Assay of Nitrotyrosine Levels

Blood nitrotyrosine levels were examined by the Cell Biolabs OxiSelect Nitrotyrosine Elisa Kit (Cat# STA-305; San Diego, California) according to the manufacturer's protocol. Briefly, mouse plasma was collected and incubated with anti-nitrotyrosine antibody and the diluted Secondary Antibody-Enzyme Conjugate. After adding the Substrate Solution and the enzyme reaction stop solution, the absorbance was measured immediately at 450 nm in a plate reader.

### Immunoblotting Assays

Immunoblotting assays of peritoneal macrophages and lung tissue samples were performed as described previously (18, 19). The lung tissues were collected and homogenized in RIPA lysis buffer. Protein concentrations were measured by a Bicinchoninic Acid Protein Assay Kit (Cat#A53225; Thermo Fisher Scientific). The protein samples were separated by electrophoresis on SDS-PAGE and transferred to polyvinylidenedifluoride membranes. The membranes were then probed with primary and secondary antibodies. Blots were developed using a Clarity Western ECL Substrate (Cat#1705061; Bio-Rad, Hercules, California).

### Statistical Analysis

Data are presented as mean ± SEM. ANOVA and *post hoc* multiple comparison tests were utilized for multiple groups. The Student's *t*-test was applied for comparisons of two groups. Data are considered statistically significant with a *P*-value < 0.05.

## Results

**Figure 1:**
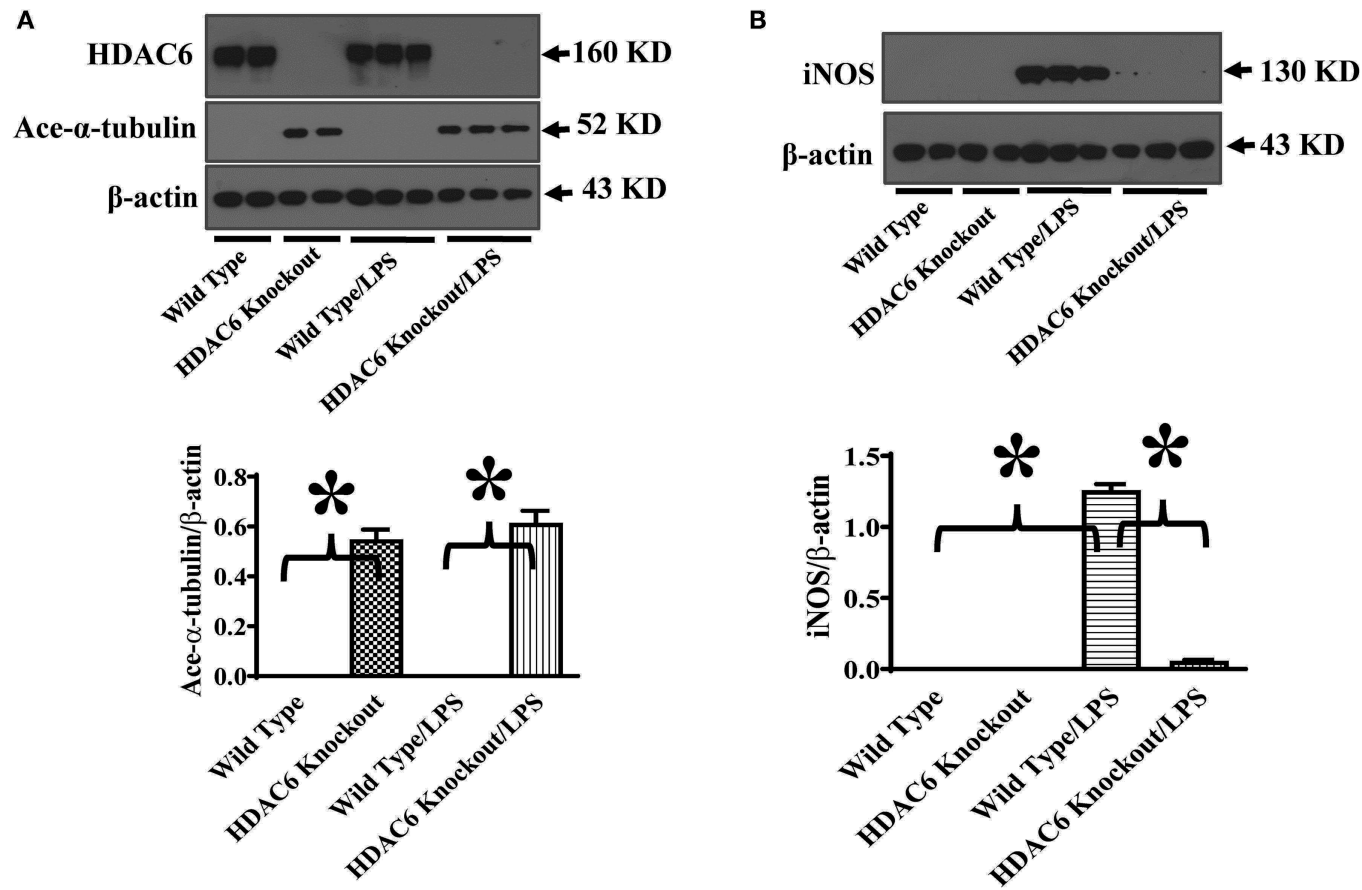
HDAC6 deletion induces robust α-tubulin acetylation and blocks LPS-induced iNOS expression in peritoneal macrophages. Peritoneal macrophages isolated from the HDAC6 Knockout and wild type mice were challenged without or with 100 ng/ml LPS for 12 h. Experiments were repeated three times. Representative blots and densitometry analysis of α-tubulin acetylation (ace-α-tubulin) **(A)** and iNOS expression **(B)**. *P < 0.05.

To investigate the role of HDAC6 in nitric oxide production, we first examined the effects of HDAC6 deletion on LPS-induced iNOS expression in macrophages. Peritoneal macrophages isolated from the HDAC6 knockout and wild type mice were challenged with LPS. Our data showed that HDAC6 deletion markedly blocked LPS-induced iNOS expression in the peritoneal macrophages, which was associated with a robust increase of α-tubulin acetylation in the peritoneal macrophages of HDAC6 knockout mice (Figure 1).

**Figure 2:**
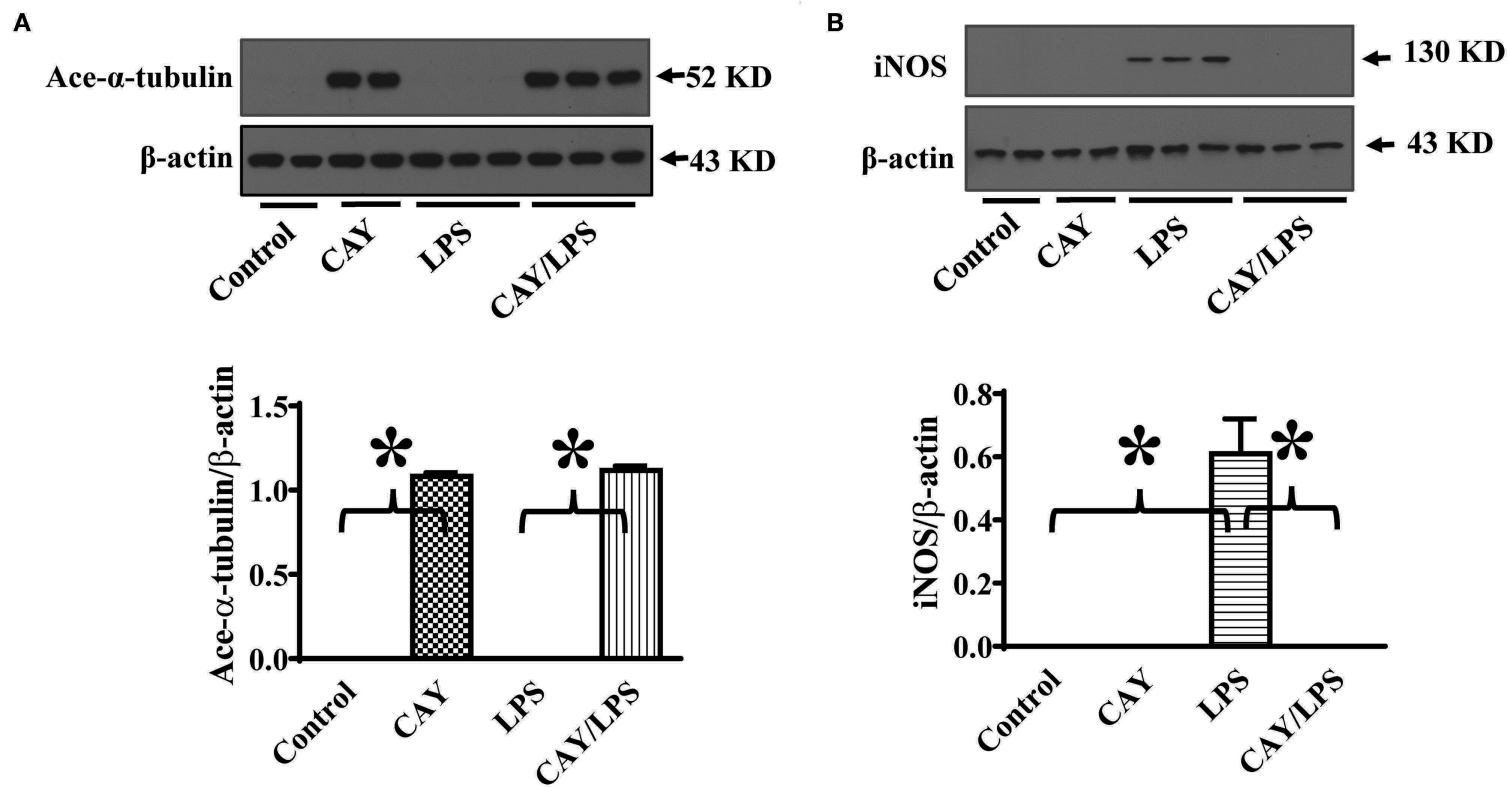
HDAC6 inhibition induces robust α-tubulin acetylation and blocks LPS-induced iNOS expression in peritoneal macrophages. Peritoneal macrophages isolated from the wild type mice were treated with 0.3 μM CAY10603 or control vehicle for 3 h. The macrophages were then challenged without or with 100 ng/ml LPS for 12 h. Experiments were repeated three times. Representative blots and densitometry analysis of α-tubulin acetylation (ace-α-tubulin) **(A)** and iNOS expression **(B)**. *P < 0.05.

To further establish HDAC6 as a target to control iNOS expression, we also conducted experiments to examine the effects of HDAC6 inhibition on LPS-induced iNOS expression in macrophages. CAY10603 is a highly selective and potent HDAC6 inhibitor with IC_50_ at 2 pM in a cell-free assay and >200-fold selectivity over other HDACs (19, 20). Peritoneal macrophages isolated from the wild type mice were pre-treated with CAY10603, then challenged with LPS. Consistent with the effects of HDAC6 deletion, HDAC6 inhibition by CAY10603 also induced a robust increase of α-tubulin acetylation and blocked LPS-induced iNOS expression in the peritoneal macrophages isolated from the wild type mice (Figure 2).

**Figure 3:**
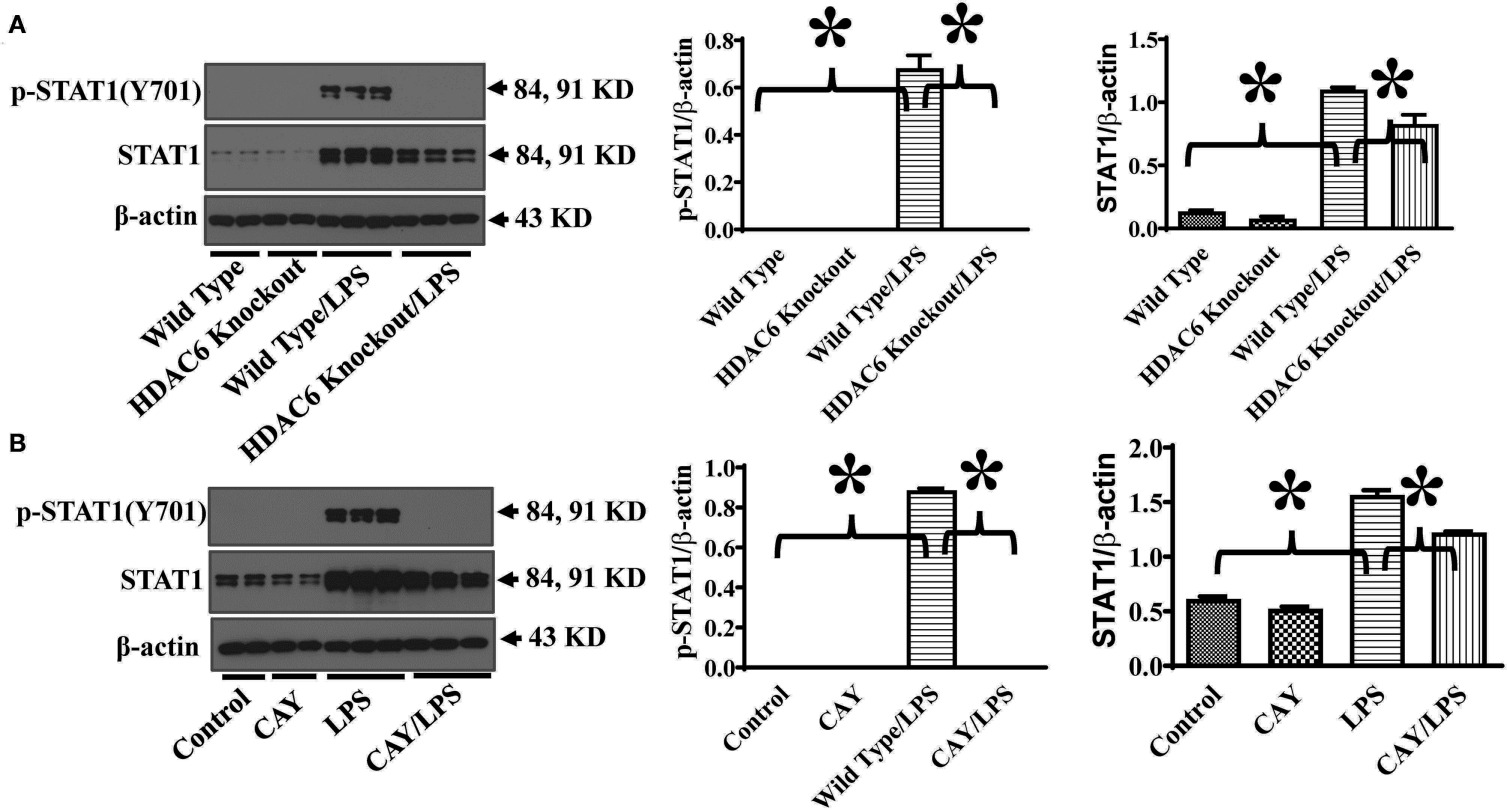
HDAC6 deletion and inhibition block LPS-induced STAT1 activation in peritoneal macrophages. **(A)** Peritoneal macrophages isolated from the HDAC6 Knockout and wild type mice were challenged without or with 100 ng/ml LPS for 12 h. **(B)** Peritoneal macrophages isolated from the wild type mice were treated with 0.3 μM CAY10603 or control vehicle for 3 h. The macrophages were then challenged without or with 100 ng/ml LPS for 12 h. Experiments were repeated three times. Representative blots and densitometry analysis of STAT1 phosphorylation at Tyr701. *P < 0.05.

HDAC6 modulates several signaling pathways through protein deacetylation (14–16). STAT1 activation is needed for maximal transcriptional up-regulation of iNOS expression (13). STAT1 phosphorylation at Tyr701 mediates STAT1 dimerization, nuclear translocation, and DNA binding (21). To investigate the underlying molecular mechanisms of HDAC6 regulation of iNOS expression, we examined the effects of HDAC6 deletion on LPS-induced STAT1 activation in macrophages by examining STAT1 phosphorylation at Tyr701. Our results showed that HDAC6 deletion blocked LPS-induced STAT1 activation as evidenced by the reduction of STAT1 phosphorylation at Tyr701 (Figure 3A). Furthermore, HDAC6 inhibition by CAY10603 showed potent inhibitory effects on LPS-induced STAT1 activation in the peritoneal macrophages isolated from the wild type mice (Figure 3B).

**Figure 4:**
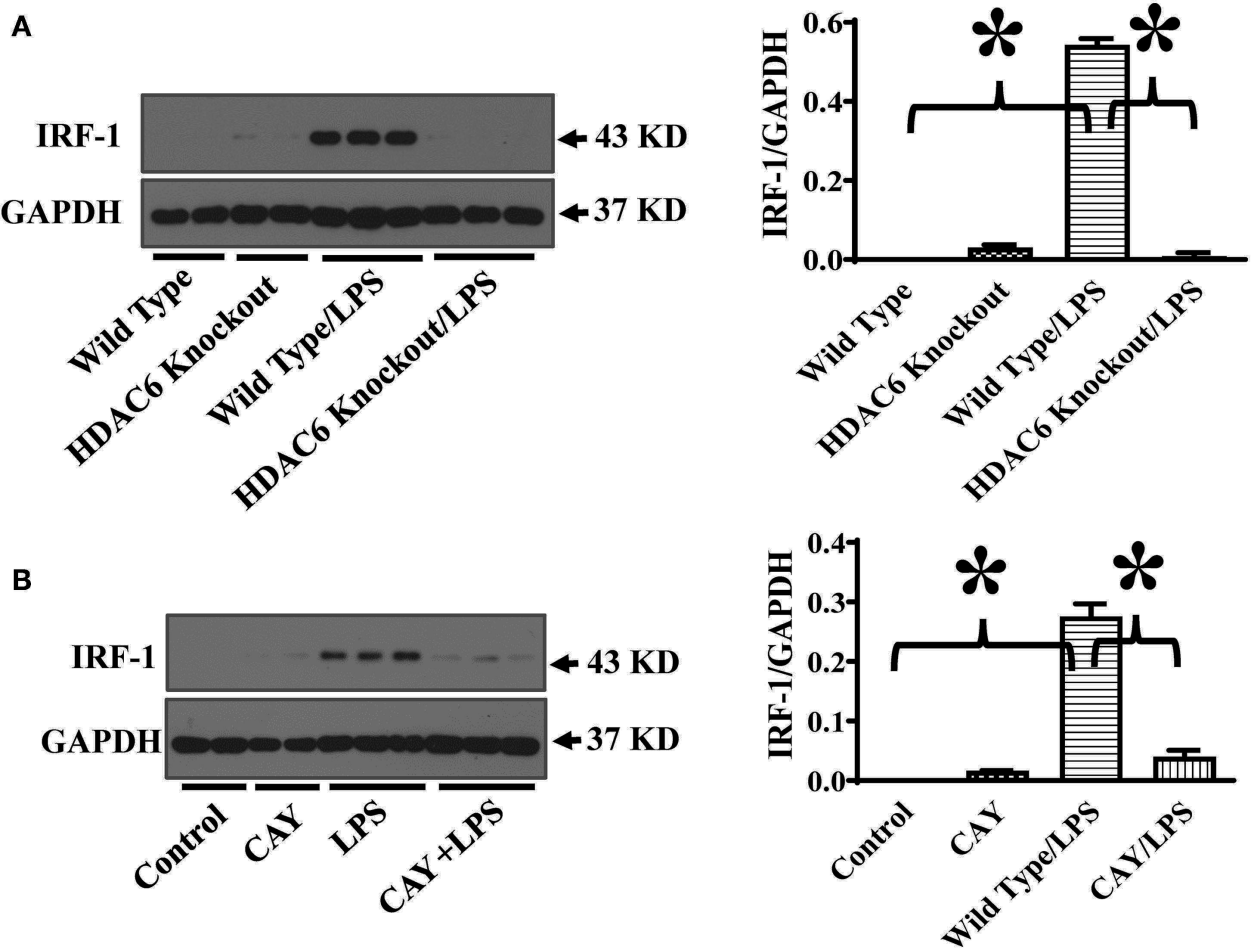
HDAC6 deletion and inhibition block LPS-induced IRF-1 expression in peritoneal macrophages. **(A)** Peritoneal macrophages isolated from the HDAC6 knockout and wild type mice were challenged without or with 100 ng/ml LPS for 12 h. **(B)** Peritoneal macrophages isolated from the wild type mice were treated with CAY10603 0.3 μM or control vehicle for 3 h. The macrophages were then challenged without or with LPS 100 ng/ml for 12 h. Experiments were repeated three times. Representative blots and densitometry analysis of IRF-1 expression. *P < 0.05.

STAT-1 activation induces the expression IRF-1 (11–13). IRF-1 promotes iNOS expression by binding to the interferon-regulatory binding site at the iNOS promoter (11–13). To further investigate the mechanisms of HDAC6 regulation of STAT1 activation on iNOS expression, we also examined the effects of HDAC6 deletion on LPS-induced IRF-1 expression in macrophages. Consistent with the inhibitory effects on STAT1 activation, HDAC6 deletion blocked LPS-induced IRF-1 expression in the peritoneal macrophages (Figure 4A). HDAC6 inhibition also showed potent inhibitory effects on LPS-induced IRF-1 expression in the peritoneal macrophages isolated from the wild type mice (Figure 4B).

**Figure 5:**
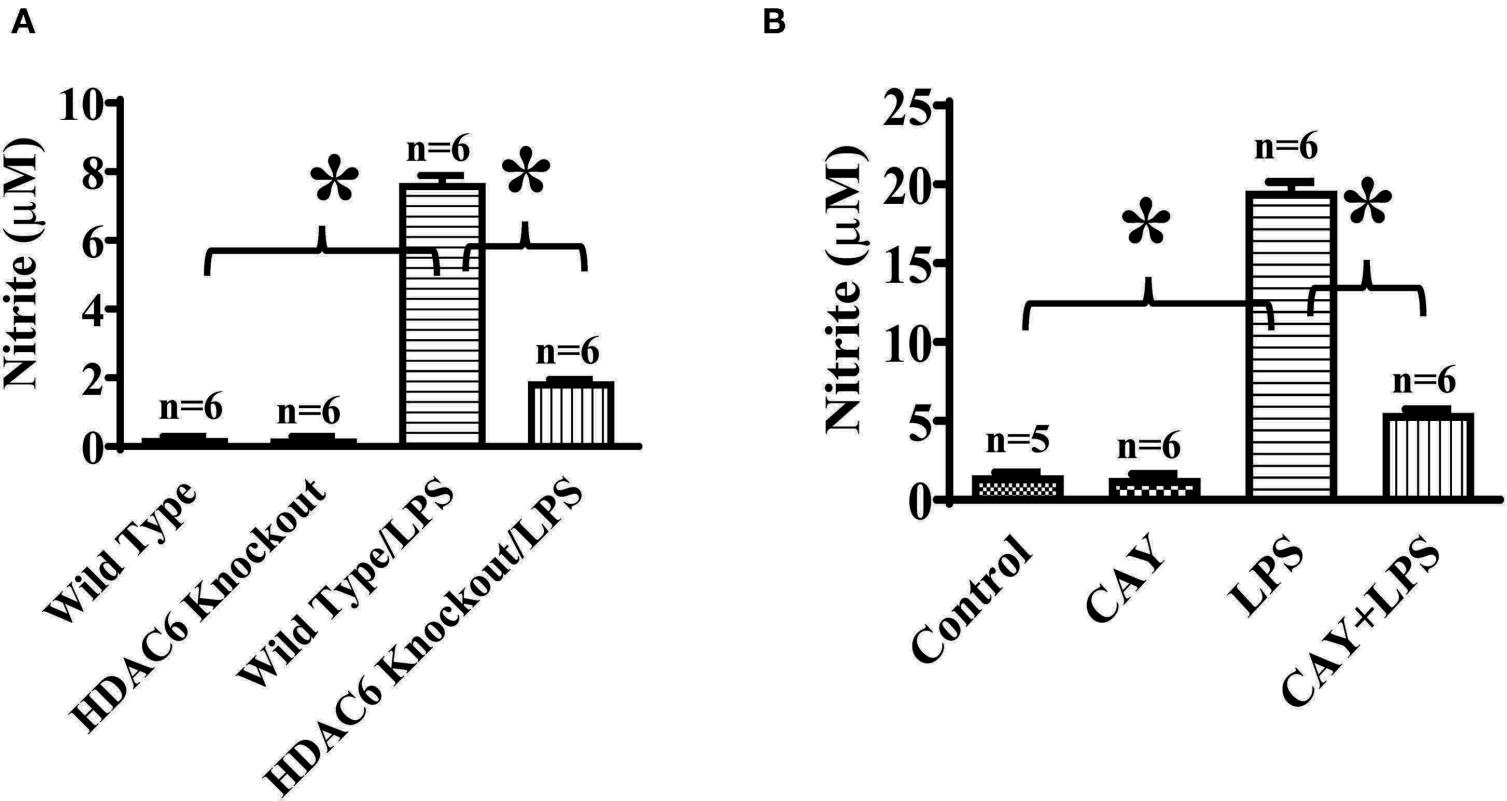
HDAC6 deletion and inhibition suppress LPS-induced nitric oxide production in peritoneal macrophages. **(A)** Peritoneal macrophages (150,000 cells/well) isolated from the HDAC6 knockout and wild type mice were challenged without or with 100 ng/ml LPS for 12 h. **(B)** Peritoneal macrophages (325,000 cells/well) isolated from the wild type mice were treated with 0.3 μM CAY10603 or control vehicle for 3 h. The macrophages were then challenged without or with 100 ng/ml LPS for 12 h. Nitric oxide production in peritoneal macrophages was assessed by nitrite quantitation. *P < 0.05.

To directly assess the role of HDAC6 on nitric oxide production, we conducted experiments to examine the effects of HDAC6 deletion and inhibition on LPS-induced nitric oxide production in macrophages. The peritoneal macrophages isolated from the wild type and HDAC6 knockout mice were challenged with LPS, nitric oxide production was examined by nitrite quantitation of cell culture supernatant. Our data indicate that HDAC6 deletion markedly reduced LPS-induced nitric oxide production in the peritoneal macrophages (Figure 5A). LPS-induced nitric oxide production was also decreased in the peritoneal macrophages isolated from the wild type mice after the pre-treatment with the HDAC6 inhibitor CAY10603 (Figure 5B).

**Figure 6:**
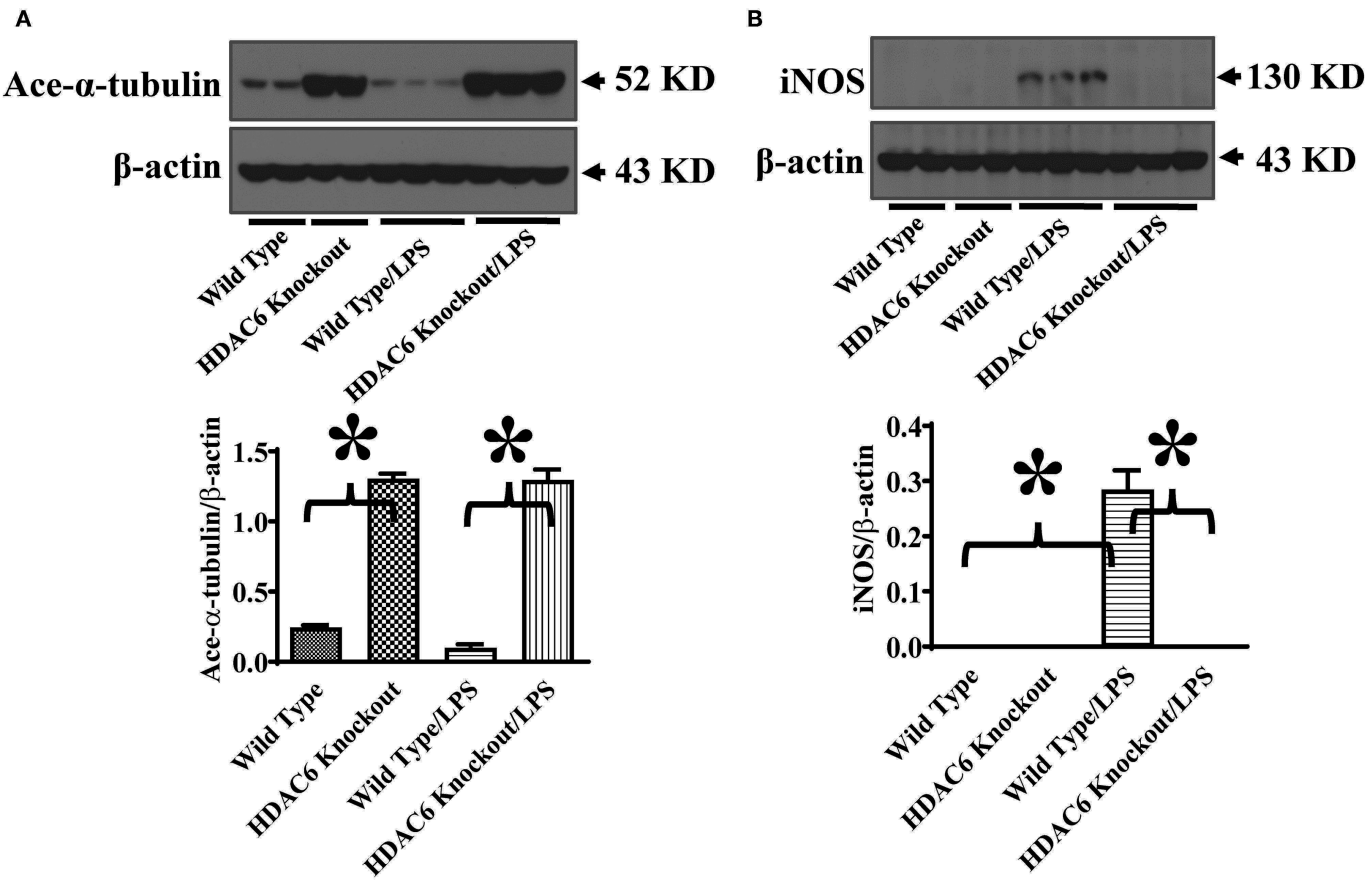
HDAC6 deletion induces robust α-tubulin acetylation and blocks iNOS expression in the lung tissues during endotoxemia. The HDAC6 knockout mice and wild type mice were divided into four groups: Wild Type (Wild type mice treated with PBS, *n* = 6); HDAC6 knockout (HDAC6 knockout mice treated with PBS, *n* = 6); Wild Type/LPS (Wild type mice challenged with LPS, *n* = 9), and HDAC6 knockout/LPS (HDAC6 knockout mice challenged with LPS, *n* = 9). The mice were challenged with LPS. 6 h after LPS challenge, lung tissues were collected. Representative blots and densitometry analysis of α-tubulin acetylation (ace-α-tubulin) **(A)** and iNOS expression **(B)**. *P < 0.05.

We then investigated HDAC6 regulation of iNOS expression during septic shock. Septic shock was induced by endotoxin. iNOS expression in the lung tissues was examined by immunoblotting assays in the wild type and HDAC6 knockout mice. Our results showed that HDAC6 deletion led to a robust increase of α-tubulin acetylation in the lung tissues, which was associated with a potent blockade of iNOS expression in the lung tissues (Figure 6). Our data establish an important role of HDAC6 in the regulation of iNOS expression during septic shock.

**Figure 7:**
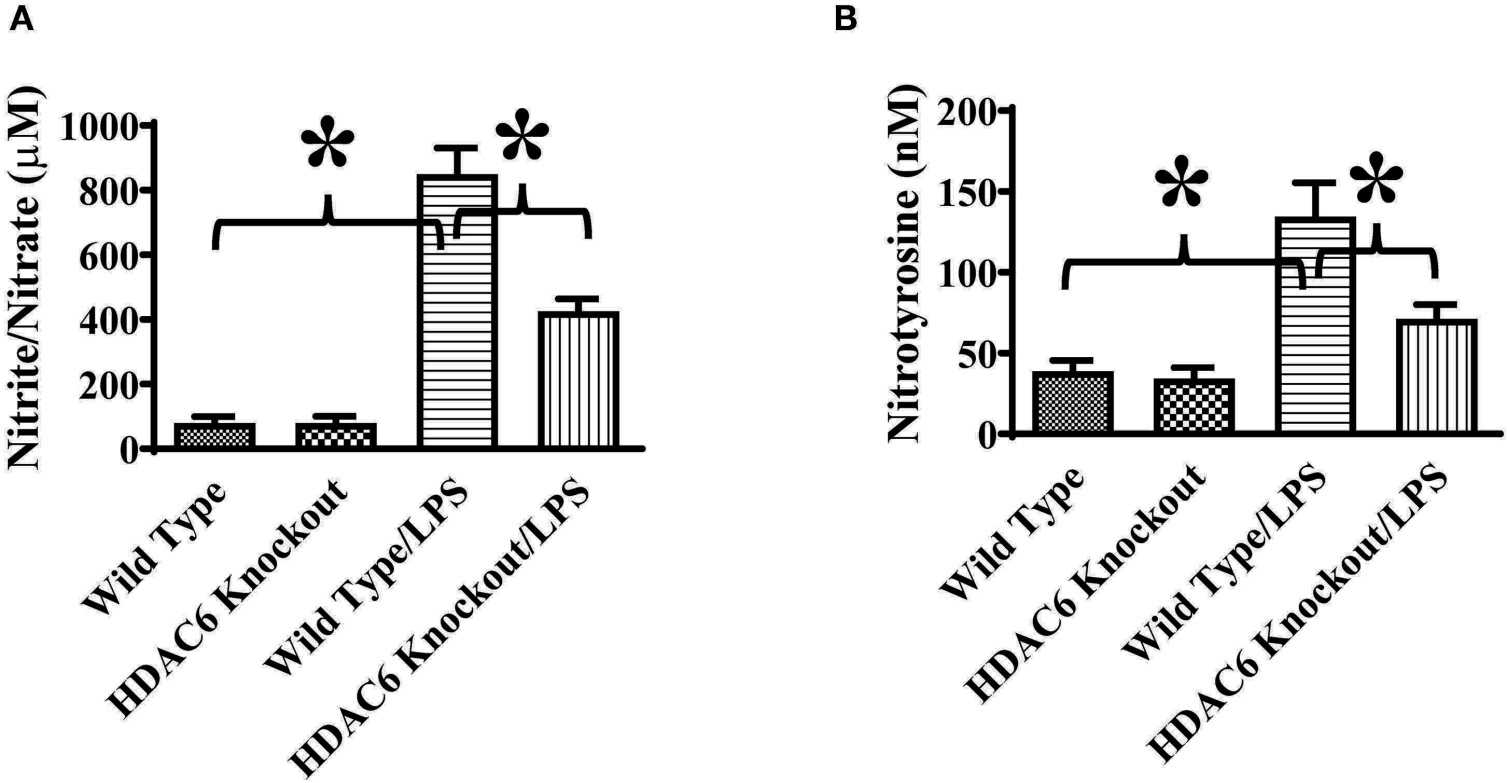
HDAC6 deletion alleviates nitric oxide over-production and nitrative stress in the blood during endotoxemia. The HDAC6 knockout mice and wild type mice were divided into four groups: Wild Type (Wild type mice treated with PBS, *n* = 6); HDAC6 knockout (HDAC6 knockout mice treated with PBS, *n* = 6); Wild Type/LPS (Wild type mice challenged with LPS, *n* = 8), and HDAC6 knockout/LPS (HDAC6 knockout challenged with LPS, *n* = 8). The mice were challenged by LPS. 6 h after LPS challenge. **(A)** Blood nitric oxide levels were examined by nitrite/nitrate quantitation. **(B)** Blood nitrotyrosine levels were examined by ELISA. *P < 0.05.

We also assessed the role of HDAC6 in excessive nitric oxide production and NO-mediated nitrative stress during septic shock induced by endotoxin. Nitric oxide levels in the blood of the wild type and HDAC6 knockout mice were examined by nitrite/nitrate quantitation. Our data showed that HDAC6 deletion significantly reduced nitric oxide levels in the blood during septic shock (Figure 7A). Furthermore, nitrotyrosine levels in the blood, an indicator of NO-mediated nitrative stress, were measured by ELISA. Our results showed that HDAC6 deletion led to a decreased blood nitrotyrosine level during septic shock (Figure 7B).

## Discussion

Dysregulated HDAC6 activity and expression have been reported to play an important role in the development of human diseases (14–16, 19). HDAC6 inhibition is a new therapeutic strategy against inflammation, cancer, and neurological disorders (14–16, 19). In our studies, HDAC6 deletion reduced the production of NO in endotoxemia, which is associated with decreased nitrative stress as evidenced by the reduced nitrotyrosine level in the blood. Our data suggest that HDAC6 can modulate NO production and NO-mediated nitrative stress during septic shock.

Excessive NO production is a major factor in vascular dysfunction and tissue damage during septic shock (6–9). Nitrite and nitrate levels in the blood are elevated in sepsis and septic shock patients (6–9). Lowering NO production has been reported to help restore blood pressure and improve vascular responses to vasoconstrictors (6–9). The decreased NO production by HDAC6 deletion and inhibition could be beneficial to control vascular responses in septic shock. Furthermore, the level of NO-generated cytotoxic peroxynitrite is markedly increased during septic shock (6–10). The nitrosylation of proteins by peroxynitrite can also alter signal transduction (6–10). Our data suggest that HDAC6 contributes to the increased peroxynitrite levels in endotoxemia, which could induce nitrative stress-mediated cellular responses and tissue injury in septic shock.

HDAC6 and iNOS have been reported to modulate inflammatory and immune responses in leukocytes (8, 14, 22–24). However, the interactions of the HDAC6 and iNOS have not been studied. Our results suggest that HDAC6 is critical for iNOS expression in leukocytes. Given the important contribution of NO in leukocyte immune responses and cytotoxic activity (8, 14, 22, 23), HDAC6 regulation of iNOS expression could affect many cellular responses of leukocytes. More studies are needed in the future to further explore these responses.

Up-regulation of iNOS expression and the resultant high NO production have been known to contribute to the development of cardiovascular diseases (6–10, 12). In pathophysiological settings, induction of iNOS is associated with detrimental effects such as life-threatening hypotension and cytotoxicity (6–10, 12). iNOS expression is induced during endotoxemia at both mRNA and protein levels (6–10, 12). Leukocytes such as macrophages are major cell types with iNOS induction and contribute significantly to the NO over-production in sepsis (6–9). iNOS-deficient mice exhibited alleviated hypotension and reduced early mortality during endotoxemia (25), suggesting that iNOS over-expression could lead to vascular dysfunction and tissue injury in septic shock. Our data provide direct evidence that HDAC6 is a key regulator of endotoxin-induced iNOS expression in macrophages and iNOS expression in tissues during endotoxemia. Our findings provide a new approach to intervene iNOS expression in leukocytes, which could improve the outcomes of septic shock such as hypotension and vascular hyporeactivity.

HDAC6 is cytoplasmic deacetylase that can deacetylate a variety of endogenous substrates such as tubulin (14–16, 19, 26). The regulation of tubulin acetylation/deacetylation states by HDAC6 could modulate microtubule-mediated biological responses, cellular function and signaling (14–16, 19). Microtubule and tubulin can bind to JAK (Janus tyrosine kinases) and STAT1 to facilitate the activation and nuclear translocation of STAT1 (27). HDAC6 has been reported to regulate α-tubulin acetylation/deacetylation and microtubule stabilization (28), indicating that HDAC6 is a potential modulator of microtubule-mediated STAT1 signaling. Our results showed that HDAC6 is need for LPS-induced STAT1 activation. Both HDAC6 deletion and inhibition led to potent inhibition of STAT1 activation and α-tubulin acetylation. Our data reveal a new HDAC6-regulated STAT1 signaling pathway, likely through α-tubulin and microtubule modification.

The role of HDAC6 in macrophage function and NO production remains large unknown. Our data suggest that HDAC6 is a key mediator of STAT1 activation, iNOS expression, and nitric oxide production in macrophages. Our findings unveil a new HDAC6-regulated cell signaling and iNOS expression pathway in macrophages. Furthermore, our results indicate that HDAC6 mediates iNOS expression in endotoxin-induced septic shock. HDAC6 could be a potential therapeutic target to control iNOS expression in septic shock.

In Summary, our results suggest that HDAC6 is a key mediator of NO over-production in endotoxemia. HDAC6 regulates LPS-induced iNOS expression in macrophages by modulating STAT1 activation and IRF-1 expression, and facilitates iNOS expression in the tissues during endotoxemia. HDAC6 inhibition could be a new therapeutic approach to control NO level and nitrative stress during septic shock, which could improve vascular responses and alleviate tissue injury in septic shock.

## Data Availability Statement

All datasets presented in this study are included in the article/supplementary material.

## Ethics Statement

The animal study was reviewed and approved by The Institutional Animal Care and Use Committee, the University of Kentucky.

## Author Contributions

YW designed the study, performed the experiments, edited the manuscript, and analyzed the data. KW designed the study, analyzed the data, and edited the manuscript. JF designed the study, analyzed the data, and wrote the manuscript. All authors contributed to the article and approved the submitted version.

## Conflict of Interest

The authors declare that the research was conducted in the absence of any commercial or financial relationships that could be construed as a potential conflict of interest.
